# Dietary Fructooligosaccharides Effectively Facilitate the Production of High-Quality Eggs via Improving the Physiological Status of Laying Hens

**DOI:** 10.3390/foods11131828

**Published:** 2022-06-21

**Authors:** Uchechukwu Edna Obianwuna, Xin-Yu Chang, Jing Wang, Hai-Jun Zhang, Guang-Hai Qi, Kai Qiu, Shu-Geng Wu

**Affiliations:** 1 Institute of Feed Research, Chinese Academy of Agricultural Sciences, Beijing 100081, China; obinogodie@gmail.com (U.E.O.); 82101202358@caas.cn (X.-Y.C.); wangjing@caas.cn (J.W.); zhanghaijun@caas.cn (H.-J.Z.); qiguanghai@caas.cn (G.-H.Q.); 2 National Engineering Research Center of Biological Feed, Chinese Academy of Agricultural Sciences, Beijing 100081, China

**Keywords:** prebiotics, fructooligosaccharides, laying performance, albumen quality

## Abstract

The focus of this study was to investigate the influence of prebiotics, such as fructooligosaccharides (FOS), on laying performance, egg quality, apparent fecal amino acid digestibility, jejunal morphology, hematological indices, immunological response, and antioxidant capacity in laying hens. A total of 216 healthy Hy-Line Brown laying hens aged 30 weeks were randomly assigned to one of three dietary treatments: basal diet, basal diet supplemented with 0.3 percent FOS, or 0.6 percent FOS. For 84 days, each treatment was fed the corresponding experimental diet. According to the findings, dietary supplementation with FOS enhanced laying performance and egg mass while lowering mortality rate. Albumen height, thick albumen content, Haugh unit, and eggshell thickness were also improved by the prebiotics. Prebiotics also boosted antioxidant status by increasing the activity of antioxidant enzymes, improved morphological development of the jejunum as demonstrated by significant increases in villi height, villi width, ratio of villi height to crypt depth, and reduced crypt depth. The prebiotics group showed a considerable increase in immunoglobulin M, G, and A (IgM, IgG, and IgA) levels, as well as a similar effect on complement proteins (C3). Furthermore, the apparent fecal amino acid digestibility of most essential amino acids was significantly enhanced. Conclusively, fructooligosaccharides at inclusion level of 0.6% efficiently enhanced laying performance and production of high-quality eggs while positively modulating amino acid digestibility, jejunal morphology, antioxidant status, and immune functions of the laying hens.

## 1. Introduction

Eggs are a vital part of human diets; thus, contribute to human nutrition due to their nutritive content, better bioavailability, and protein digestibility [1]. Not only are whole eggs consumed as food but the use of liquid egg products such as albumen, owing to its technological properties as functional food, raw materials for health and the food processing industry has gained research interest in recent times. Bioactive peptides of albumen proteins have gained attention due to their application for human drugs [2,3], controlling astringency in wines [4], improvement in food texture [5], and as antimicrobial agents for food safety [6,7]. Additionally, albumen of strong elasticity and thick albumen content extends shelf life of eggs [8]. All these highlight the crucial need to produce eggs of high-albumen quality for human benefit. 

Production of high-quality eggs may reflect the capacity of laying hens to absorb and utilize nutrients in response to a change in feeding strategies. Laying hens are prone to oxidative stress and immunity challenges due to the environment and high metabolic demand owing to egg production [9]. In order to meet up with the challenging task of improving egg production, egg quality, and animal health, farmers often adopt the application of synthetic antimicrobials and growth enhancers [10]. However, eggs are one of the most commonly consumed animal proteins; thus, use of antibiotics may produce eggs that are not healthy or safe due to contamination with antibiotic residues [11]. Hence, production of high-quality eggs that are safe is achieved with adoption of nutritional strategies because diets contain various nutrients for maintenance and production in laying hens. This has harnessed the exploration of other growth-enhancing and immune-strengthening alternatives, as natural safe feed additives and total replacement for antibiotics [12]. The use of prebiotics as a safe feed additives and replacement for antibiotics in poultry production has gained attention [13].

Prebiotics as feed additives are considered as non-digestible oligosaccharides, which serve as a fermentable substrate to enhance the growth of beneficial microflora and probably function as competitive adhesion sites for pathogenic bacteria [14]. Therefore, prebiotics can be utilized in the livestock sector as safe feed additive to improve animal health and performance [15]. Most commonly used prebiotics in poultry industry are: xylooligosaccharides (XOS), mananoligosaccharides (MOS), chitooligosaccharides (COS), and fructooligosaccharides (FOS). Fructooligosaccharides are resistant to enzymatic degradation, while being absorbed in the upper gut and they enters into the caecum where most of the fermentation process occurs in avian species, probably due to their possession of β-linkages [16]. The fermentation of most prebiotics in the gut and their capacity to create a favorable microecological environment in the gut may account for their effect on laying performance [17].

Eggs are the main output in the laying hen industry; egg production rate and quality determine production efficiency and economic returns. Various reports have shown that prebiotics improve egg production and performance [14,18,19], although some studies reported no effect of prebiotics on egg production [20,21]. In same line, egg quality reflects the internal (albumen and yolk) and external components (shell) of eggs which must be maintained to enhance the availability of functional food ingredients for the food industry. Previous studies reported that dietary prebiotics improved shell quality [19,20] and albumen quality [18,22,23] in laying hens, but some showed no effect on shell thickness [24] and Haugh unit [20]. 

Furthermore, production of high-quality eggs, a reflection of both external and internal components of the egg, is orchestrated by physiological processes which hinges on nutrient utilization and animal health. Disruptions in animal health and metabolic status often result in: alteration in the quaternary and secondary structure of the albumen protein [25], cause gut dysfunction and render the animal vulnerable to pathogen invasion [17], and may impair the oviduct health [26] leading to poor laying performance and egg quality. Prebiotics have the potential to protect host immunity, mitigate oxidative stress, improve intestinal health and, consequently, egg production and egg quality [14,19,27,28]. Therefore, improving the physiological status of the birds as a boost to nutrient utilization in laying hens is essential for production of high-quality eggs.

It becomes expedient to investigate the possible role of prebiotics in improving albumen quality. The evidence remains rather limited with respect to the dosage and the underlying mechanism for effect on egg quality in laying hens. For this reason, the present study sought to investigate the beneficial potential of FOS as feed additives at different inclusion levels on performance, egg quality, intestine health, amino acid digestibility, blood and serum parameters of laying hens.

## 2. Materials and Methods

### 2.1. Ethics Statement

All the experimental procedures were approved by the Animal Care and Use Committee of Feed Research Institute of the Chinese Academy of Agricultural Sciences, Beijing. The approval number of animal ethics is CAAS.No20200507S0600103. 

### 2.2. Experimental Design and Bird Management

A total of 216 Hy-Line Brown laying hens (30 weeks old, egg production rate = 89.0 ± 1.5%) were randomly assigned to one of three treatment groups, each consisting of 6 replicates and 72 hens. In total, 12 birds in four neighboring cages were considered as a replicate with three birds housed in one cage (40 40 35 cm) fitted with a nipple drinker and a trough feeder. The hens were given ad libitum feed and water, and they were vaccinated and handled according to the Hy-line International Online Management Guide (Hy-Line International, West Des Moines, IA, USA, 2011). The laying hens were fed corn-soybean laying hen diets in a mash form (basal diet) prior to the experiment and the diet was of standard and adequate in all nutrients. The basal diet was formulated to meet the nutrient requirements of the National Research Council (NRC, 1994) and Chinese Feeding Standard of Chicken (NY/T, 33-2004), and its ingredient composition and nutrient levels are presented in Table 1. The control group was provided a basal diet without prebiotics supplementation. The two treatment groups were offered the basal diet supplemented with either 0.3% or 0.6% FOS, respectively. The fructooligosaccharides were provided by Cofco Nutrition and Health Research Institute, China. The experiment was performed over 14 weeks (30–44 weeks of age) consisting of 2 weeks of an acclimation or feed transition period and 12 weeks of experimentation.

On a daily basis, the controlled pen house environment comprised 16 h of light, a temperature of 24 °C, and a humidity of 50–80%. Throughout the trial, the animals’ health was stable, and there was no outbreak of disease of any kind. During the 12-week feeding trial, daily records of egg number and weight, as well as the mortality rate per replicate, were kept, whereas feed intake was reported biweekly. From the recorded data, we estimated average feed intake (ADFI), hen day production (HDP), average egg weight (AEG), egg mass, and feed conversion ratio (FCR) for the entire study period.

### 2.3. Sample Collection

On the last day of each of the four weeks (4, 8, and 12), 18 eggs were retrieved from each treatment group (three eggs from each replicate with a weight that was near to the replicate range). After a 12 h fast, 24 birds (six from each group, one per replication) were chosen for sample collection at the end of the study period (12th week). BD Vacutainer^®^ Plus Plastic Serum Tubes (Becton Dickinson, NJ, USA) and BD Vacutainer^®^ Plus Plastic K2EDTA Tubes (Becton Dickinson, NJ, USA) were used to collect about 5 mL of blood from the jugular veins for determination of serum and whole blood parameters, respectively. To obtain serum, blood samples collected in vacutainer tubes were placed in a slanting posture for 30 min before being centrifuged at 300× *g* for 10 min at 4 °C and stored in 1.5 mL Eppendorf tubes at −20 °C. Pentobarbital sodium (100 mg/kg BW) was used to euthanize the birds, which were then cut up under aseptic conditions. The small intestine of each bird was removed and placed on ice, while the heart, liver, magnum, and spleen were removed, weighed, and their relative weight computed as weight of organ (g)/body weight (g) × 100%. Their mesentery was also cleaned and flattened, and about three centimeters of jejunum (medial portion posterior to the bile ducts and anterior to Meckel’s diverticulum) were collected, washed in physiological saline solution, fixed in 10% buffered formalin, and stored at 4 °C for histology examination.

### 2.4. Egg Quality Assessment

The 18 eggs collected from each group were kept under normal room temperature and analyzed within 1 h of collection. The eggs were broken, and the albumen and yolk were divided using an egg separator and respectively weighed. The eggshells were washed of albumen residues and naturally air-dried for 48 h before being weighed. The ratio of albumen, yolk, and shell was calculated as their weight/egg weight × 100. Eggshell thickness was measured using an Eggshell Thickness Gauge (average of three sites around the eggshell: air cell, equator, and sharp edge) (ESTG-1, ORKA Technology Ltd., Ramat HaSharon, Israel). An Egg Force Reader was used to measure the breaking strength of eggshells (ORKA Technology Ltd., Ramat HaSharon, Israel). An automated Egg Analyzer was used to detect albumen height, Haugh unit, and yolk color readings (ORKA Food Technology Ltd., Ramat HaSharon, Israel). Further egg quality testing was undertaken by putting the weighed albumen through a 60-mesh sieve and allowing it to pass for 30 s. The thick component of the albumen remained stuck to the sieve and was weighed as thick albumen, whilst the filtrate was weighed as thin albumen. 

### 2.5. Histology and Jejunal Morphometric Analysis

The fixed jejunal samples were cleaned, dehydrated, clarified, and paraffin embedded. Then, they were cut into 6-mm-thick pieces, mounted on glass slides, dewaxed using xylene, hydrated, and stained with hematoxylin and eosin. Images were examined and taken using a microscope (an Olympus BX43 microscope; Olympus Corp., Tokyo, Japan). All regents utilized in the histology evaluation were of analytical quality (Sinopharm Chemical Reagent Co., Ltd., Beijing, China). In terms of morphometric indices, intact villi (*n* = 10) and corresponding crypts were chosen to measure villus height (VH: measured from the top of the villus to the villus–crypt junction), crypt depth (CD: measured from the base up to the crypt–villus transition region), and villus width (VW—at the middle point of the villus). Data from villi height and crypt depth was used to obtain VH:CD ratio and villi surface area (VSA) was calculated using 2π × (VW/2) × VH [29].

### 2.6. Determination of Hematological and Serum Indices

Within 1 h of collection, blood was drawn in an EDTA tube and transported to the laboratory for hematology testing. An Auto Haematology Analyzer was used to examine hematological indices (Model: BC-2800 Vet). MCH, MCV, and mean cell hemoglobin concentration (MCHC) were calculated as red blood cell indices. For the determination of serum parameters, the serum was thawed at 4 °C before analysis and kept at a low temperature throughout the experiment to avoid enzyme activation. The activities of glutathione transferase (GST), glutathione peroxidase (GSH-Px), superoxide dismutase (T-SOD), catalase (CAT), total antioxidant capacity (T-AOC), and MDA content in the serum were measured spectrophotometrically and assayed using chicken enzyme-linked immunosorbent assay (ELISA) kits with catalogue numbers: (ml023160, ml061730, A001-1-1, A007-1-1, ml063644 and A003-1). ML Bio and Nanjing Jiancheng Bioengineering Institute, (Nanjing, China) was the source for all kits. The concentrations were expressed in micromoles per milliliter of serum (CAT, T-SOD, T-AOC), nanograms per milliliter of serum (GST, GSH-Px), and nanomoles per milliliter of serum (MDA). Using a microplate reader and the appropriate ELISA kit, the contents of immunoglobulins IgA, IgG, IgM, and complement proteins C3 and C4 in the serum were determined (WLB-091301, WLB-050501, WLB-09120, E032-1-1 and E033-1-1). Immunity index concentrations were measured in micrograms per milliliter of serum (IgG, IgM), nanograms per milliliter of serum (IgA), and milligrams per milliliter of serum (IgA) (C3 and C4). The study’s ELISA assays all have great sensitivity and specificity for hens and were used according to the manufacturer’s instructions.

### 2.7. Apparent Fecal Amino Acid Digestibility

After 12 weeks, 3 birds from each replicate were chosen and placed in a metabolic cage with a feces sample collection tray. For three days, fecal samples were collected at 12 h intervals and stored in sealed bags at −20 °C. Feed, feathers, and other extraneous components in the feces samples were meticulously removed during collection to guarantee that the fecal sample was not contaminated. Fecal samples were thawed and dried at 70 °C for 72 h before being pulverized to a fine powder that could pass through a 0.05 mm mesh. The feed intake and feces weight (dry matter basis) from each metabolic cage were computed for apparent fecal amino acid digestibility. Amino acid analysis was performed on the fecal and feed samples using HPLC and adopting the established method by [30]. The HPLC system Finnigan Surveyor Plus and HyperSil BDS C18 column, size 250 × 4.6 mm, 5μm (Thermo-Electron Corporation, Waltham, MA, USA) was used. Apparent fecal amino acid digestibility % was calculated as: 1 − (amino acid concentration in feces ÷ amino acid concentration in feed) ×100.

### 2.8. Statistical Analysis

SPSS for Windows version 19.0 was used to conduct all statistical analyses (SPSS Inc., Chicago, IL, USA). The one-way Analysis of Variance (ANOVA) was applied to all data. Replicates were used as experimental units in this study. The variation in the data was expressed as pooled standard error of the mean, while the data were expressed as means. Tukey’s test was used to compare the means of dietary regimens, and results with a *p* value of 0.05 were considered statistically significant. The Principal Component Analysis (PCA) was performed to unearth the correlation structure between the analyzed samples using a Principal Component Analysis (PCA) on centered log-ratio transformed sequence counts with the zCompositions R package v1.34.

## 3. Results 

### 3.1. Performance

Table 2 summarizes the performance of the laying hens. Throughout the feeding period, dietary FOS average had no effect (*p* ≥ 0.05) on egg weight and FCR. Diets had no effect (*p* ≥ 0.05) on egg mass at the end of weeks 4 and 8 but had a significant effect (*p* ≤ 0.05) at the end of weeks 12 and 1–12. Additionally, while dietary FOS had no effect (*p* ≥ 0.05) on egg production rate at the end of week 4, there were significant increases (*p* ≤ 0.05) in the diet group compared to the control group at weeks 8, 12, and 1–12. After week 12, the egg production rates of the supplemented groups were only substantially different (*p* ≤ 0.05). In the control group, the number of damaged eggs was higher than in the experimental group but not statistically different (*p* ≥ 0.05) while zero mortality was recorded in the diet group throughout the study. Diets had no effect (*p* ≥ 0.05) on bird feed consumption at weeks 4 and 8, but substantial increases (*p* ≤ 0.05) in the treatment group compared to the control group were detected at weeks 12 and 1–12. Additionally, only at week 12 did significant differences (*p* ≤ 0.05) between the supplemented groups emerge.

### 3.2. Egg Quality

Table 3 shows the egg quality metrics of laying hens fed with prebiotics. Dietary FOS had a significant (*p* ≤ 0.05) impact on the relative albumen weight of the eggs at weeks 8, 12, and 1–12. Only toward the end of week 8 did significant differences (*p* ≤ 0.05) between the supplemented groups emerge, but not at subsequent evaluation points. Throughout the feeding trial, the relative yolk weight of the control group was considerably higher (*p* ≤ 0.05) than the supplemented group. Relative shell weight of the diet group was significantly higher (*p* ≤ 0.05) relative to the control group at week 8 but no significant changes (*p* ≥ 0.05) were identified at weeks 4, 12, and 1–12. Diets influenced (*p* ≤ 0.05) shell thickness at weeks 4 and 1–12, but no differences (*p* ≥ 0.05) were identified at weeks 8 and 12. Only at week 12 did dietary interventions have a significant effect (*p* ≤ 0.05) on yolk color. Dietary treatments significantly increased (*p* ≤ 0.05) albumen height, Haugh unit, and thick-to-thin albumen ratio at weeks 12 and 1–12 with significant variations (*p* ≤ 0.05) between the diet groups but no differences (*p* ≥ 0.05) were found at weeks 4 and 8, except for albumen height at week 8. 

### 3.3. Antioxidant Capacity and Immune Indices of Serum

Table 4 shows the dietary effects of FOS on the antioxidant and immunological capacities of laying hens. Dietary treatments substantially influenced (*p* ≤ 0.05) the activities of GSH-Px, GST, T-SOD, CAT, and MDA content in the serum, with antioxidant enzyme activities higher in the treated group and MDA content higher in the control group. On T-AOC, there was no treatment effect (*p* ≥ 0.05). Between the supplemented groups, there were significant differences (*p* ≤ 0.05) in CAT, T-SOD, GSH-Px, and GST activities, but no difference (*p* ≥ 0.05) in MDA content. Furthermore, the dietary treatment had a substantial (*p* ≤ 0.05) impact on the contents of IgA, IgG, and IgM. There were no significant variations (*p* ≥ 0.05) in IgA and IgM between the supplemented groups but variation in IgG was found. Additionally, complement protein (C3 and C4) C3 was influenced (*p* ≤ 0.05) by diets but no effect on C4 was found.

### 3.4. Hematology Indices

Table 5 shows the dietary effects of FOS on laying hen blood parameters. In comparison to the control, WBC, heterophils, and lymphocytes increased significantly (*p* ≤ 0.05) in response to nutritional treatment. In response to nutritional treatment, no significant effect (*p* ≥ 0.05) on other indicators was identified.

### 3.5. Organ Indexes and Jejunum Morphology

The relative organ weight of the heart, magnum, spleen, and liver were not influenced by treatments (data not shown). Table 6 presents the quantitative results of the jejunum villi morphology in response to the dietary treatments. The VH, VW, VSA, and V/C increased significantly (*p* ≤ 0.05) in response to dietary treatment while CD was significantly reduced by the dietary treatment and higher in the control group.

### 3.6. Apparent Fecal Amino Acid Digestibility

The apparent fecal amino acid digestibility coefficients of laying hens fed prebiotics-based diets are presented in Table 7. The digestibility of crude protein was significantly influenced (*p* ≤ 0.05) by dietary FOS and there were no variations (*p* ≥ 0.05) between the treated groups. The digestibility coefficients of essential amino acids (threonine, methionine, isoleucine, tryptophan, and lysine) and non-essential amino acids (asparagine, serine, glutamine, and glycine) were significantly higher (*p* ≤ 0.05) in the dietary treatment group compared to the control. There was no influence (*p* ≥ 0.05) of dietary treatments on the digestibility coefficients of proline, alanine, cysteine, valine, phenylalanine, and histidine. Additionally, no significant differences (*p* ≥ 0.05) in digestibility of asparagine, threonine, serine, glutamine, glycine, and methionine-cysteine were observed between T2 and T3, but only T2 differed from the control. There were also significant variations (*p* ≤ 0.05) in digestibility of methionine among T1, T2, and T3. Further analysis revealed no variations (*p* ≥ 0.05) in isoleucine, tyrosine, lysine, or tryptophan between T2 and T3; however, both were significantly different (*p* ≤ 0.05) from the control.

### 3.7. Principal Component Analysis (PCA)

**Figure 1:**
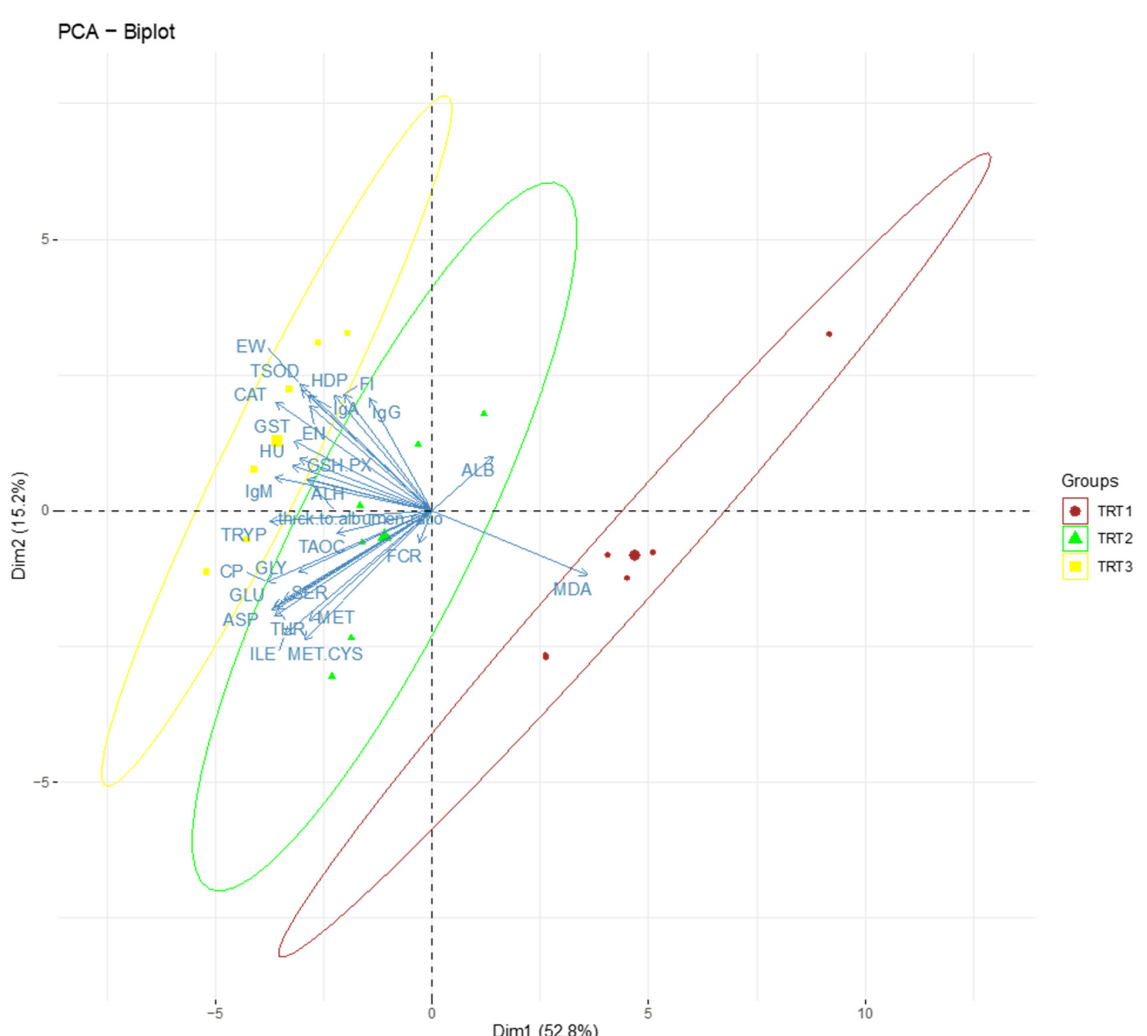
Biplot from the Principal Component Analysis of dietary treatments: Treatments 1, 2, and 3 correspond to red, green, and yellow.

The application of PCA allows for better analysis and comparison of similarities between groups by reducing the number of variables. We took into consideration a normalized version of the data to obtain a PCA biplot presentation (Figure 1.). The first component covered 52.8% and the second component covered about 15.2% of the total variance. 

## 4. Discussion

Over the decades, functional oligosaccharides have been used as prebiotics in poultry nutrition as an alternative replacement for synthetic antibiotics in enhancing performance and health of laying hens. However, no studies to date have reported the supplementation of fructooligosaccharides as prebiotics at 0.3 and 0.6% inclusion levels in laying hens; thus, owing to limited literature, we compared our findings with other studies but with same group of functional oligosaccharides. Our results showed that dietary FOS at 0.3 and 0.6% inclusion levels exerted beneficial influences on egg production and quality, intestinal morphology, apparent fecal amino acid digestibility, immune and antioxidant function. Therefore, natural additives such as fructooligosaccharides, a type of prebiotic, are considered safe in poultry nutrition. 

### 4.1. Effects of Fructooligosaccharides on Laying Performance

Previous reports have demonstrated the positive effect of dietary prebiotics including FOS on egg production rate in laying hens [19,24,27,28,31]. Additionally, there is others evidence of increased egg mass due to dietary prebiotics [19,28]. The improvements in egg production and egg mass may be associated with enhancement effects of prebiotics on nutrient utilization [32]. Our findings are in agreement with other functional oligosaccharides; egg production and egg mass were improved due to dietary FOS. Previous research has shown that egg production is often affected by animals’ health status while enhanced immune response and antioxidant capacity may aid to protect the animal from tissue oxidative damage and pathogen invasion [14,19]. Therefore, the improvement in egg production could be that animal health was boosted as evidenced by the increased activity of antioxidant enzymes and immunoglobulin secretion observed in this study. This may have protected the animal from tissue oxidative damage and supported better health status with consequent improvement in the egg formation process occurring in body of the animal. Additionally, prebiotic fermentation in the gut promotes growth of beneficial microbes and suppresses pathogens, leading to the development of improved villi structures [16,33]. The positive effect on egg production could be attributable to the increased villi structures observed in this study: the favorable gut environment due to FOS fermentation may have stimulated development of jejunal villi structures which acted as a driver for efficient nutrient utilization. Furthermore, egg production is sensitive to amino acid nutrition imbalance [34]. There is evidence that essential amino acids (methionine, threonine, isoleucine, and lysine) exerted a positive influence on egg production [35,36,37], probably because amino acids provide support for high protein synthesis which is often a prerequisite for improved egg production. Thus, the improved digestibility of these essential amino acids in this study suggests their utilization for egg production. Meanwhile, there are studies that reported no significant effect of prebiotics on egg production [20,21,38]. The inconsistent results could be due to the age of laying hens, dosage of the supplement, and components of the prebiotics used. All these findings highlight that the improvement in laying performance is adducible to the protective effect on gut integrity, marked villi structures, and improved health status which all act as a boost for amino acid digestibility and efficient nutrient utilization.

Furthermore, feed intake and feed conversion ratios are often used to depict the extent of nutrient utilization by the animals. The study of [28] reported that FOS decreased feed intake and improved FCR, whereas oligofructosaccharides enhanced feed efficiency but had no effect on feed consumption [39], in laying hens. The improved feed utilization may be linked to the capacity of prebiotics to stimulate activities of digestive enzymes such as aminopeptidase, protease, and amylase [40]. The current findings showed that dietary FOS improved feed intake but had no significant effect on FCR. FOS are often considered as sweeteners [41] and enhancers for intestinal protease activities [16]. Additionally, tryptophan acts as a precursor of serotonin, a neurotransmitter that controls feed intake [42]. Therefore, we could infer that the increased appetite may be due to enhanced enzymatic activities triggered by favorable gut environment, improved taste of the feed, and better digestibility of tryptophan. On the contrary, prebiotics had no significant effect on feed intake of birds [16,20,43]. The variations among these studies may be due to type of prebiotics and dosage level used. In addition, we observed a reduction in percentage of cracked eggs and zero-mortality rate in the feed additives group, which is similar to the findings of [20]. Respectively, these could be attributable to the beneficial effect of FOS on eggshell thickness and the improved health status of the birds evidenced by enhanced immune capacity. Thus, FOS could be used as a safe additive to improve feed intake and reduce mortality rate with consequent improvement in egg production and egg quality. 

### 4.2. Effects of Fructooligosaccharides on Egg Quality of Laying Hens

Egg components, both external (shell quality) and internal (albumen and yolk), must be maintained to meet consumer demands for functional eggs. Previous reports have shown that COS and XOS fed to laying hens enhanced eggshell thickness [19,20]; the improvement may be related to the positive influence of prebiotics on mineral absorption rate of calcium and magnesium [20,44] which enhances eggshell integrity. In the current study, there was a beneficial effect of FOS on eggshell thickness but no dietary influence on eggshell strength was found. The positive effect may be linked to FOS fermentation which stimulates proliferation of enterocytes and lowers intestinal pH which catalyzes ionization of minerals [38]. Thus, the enhanced intestinal villi structures observed in this study could improve intestinal calcium absorption and bioavailability with consequent improvement in eggshell quality. Contrary to our findings, dietary FOS, MOS, and inulin [24,28,45] had no effect on eggshell thickness. The lack of influence on eggshell quality may be due to the dosage of supplements and age of hens. Additionally, egg yolk color, which is associated with consumer preference and acceptance [46], is influenced by dietary prebiotics. We observed an increased yolk color due to dietary FOS, similar to other reports on dietary prebiotics in laying hens [18,21,22], whereas [20] reported no effect of XOS on egg yolk color. The improved yolk color and the variations may be due to prebiotic composition but were not very clear in this study. The increased yolk weight in the control group may be linked with the lower albumen proportion observed when compared to the treatment group.

Further, improved albumen quality would sustain the increased demand for liquid egg products. Dietary oligosaccharides fed to laying hens improved HU values [18,21,22,23]. The study of [22] showed that dietary COS enhanced albumen height but was not statistically different from control. The improvement in albumen quality may be linked to improved nutrient utilization resulting in better protein synthesis. Our findings are in affirmation with previous reports that prebiotics improve albumen quality. In this study, there were significant improvements in albumen quality (increased Haugh unit, albumen height, and thick-to-thin albumen ratio), in response to dietary FOS. The influence of prebiotics on thick-to-thin albumen ratio has not been previously reported. The enhanced albumen synthesis may be attributable to the health status of the animals. Prebiotics have been found to enhance ovary health by suppressing pathogens [14,17]. On the other hand, reproductive performance may decline in animals due to accumulation of free radicals which often culminates in damage of tissues and alteration of functional proteins [47]. Therefore, the enhanced immune function and antioxidant capacity observed in the study may suggest a protective effect on the reproductive tract from pathogen invasion and oxidative damage, leading to healthy tubular glands of the magnum and consequently increased synthesis of ovomucin. The β-subunit of the ovomucin contains an increased proportion of *O*-linked oligosaccharides on a repeated domain abundant in threonine and serine [48]. Additionally, it has been reported that isoleucine [49] and total sulphur amino acids (TSAA) [35] enhance albumen quality. The improvement in apparent fecal digestibility of total sulphur amino acids and isoleucine in this study may contribute to the enhanced albumen quality. It could be inferred that the improved health status and villi structures may have positively stimulated the digestibility and absorption of the amino acids; thus, high bioavailability of amino acids needed for albumen synthesis. However, XOS [20] and marine-derived prebiotics [18] had no effect on HU value and thick albumen height, respectively, while COS had no effect on both albumen indices [19]. The inconsistencies among these studies could probably be due to dosage of supplements, duration of feeding, and type of prebiotics used. Taken together, dietary FOS could be used as feed supplements in the diet of laying hens to enhance eggshell quality and content of thick albumen, which would respectively reduce the rate of cracked eggs during transportation and provide liquid egg products for consumers, food processing, and the pharmaceutical industry. Improvements in egg production rate and egg quality are often pre-determined by the physiological status of the birds.

### 4.3. Effect of Fructooligosaccharides on the Antioxidant and Immune Capacity, and Hematology of Laying Hens

The health status of the animals is often assessed based on indicators including: antioxidant capacity [50], hematological parameters [51], and serum concentrations of immunoglobulins (IgA, IgM, and IgG). It has been established that activities of antioxidant enzymes T-SOD, GSH-Px, GST, and CAT serve as the body’s first line of defense in response to elimination of reactive oxygen species [52]. Oxidative stress is often measured using MDA content, which is an oxidative biomarker and often denotes the end product of lipid peroxidation [13]. Previous reports have proven that prebiotics could cause an inverse relationship between antioxidant enzymes and oxidative biomarkers in both serum [20] and tissues [18,53] of laying hens; thus, enhancing the antioxidant capacity of the animal. Increased serum activities of T-SOD and CAT, GSH-Px, and T-SOD [18,54] were observed in birds fed marine-derived prebiotics and COS, respectively, with a consistent reduction in MDA content. In this study, the increased activity of antioxidant enzymes and reduced oxidative biomarker lend evidence once again to the inverse relationship between antioxidant enzymes and oxidative biomarkers due to the prebiotic effect. This is suggestive that FOS could improve the antioxidant capacity of laying hens by stimulating the enzymatic component of the antioxidant system relative to the non-enzymatic antioxidant system. Methionine acts as a glutathione (GSH) precursor which reduces reactive oxygen species, protecting the cells from oxidative damage [55]; therefore, significant digestibility of methionine may be a key factor for the activity of antioxidant enzymes. Thus, the antioxidant property of FOS suggests its potential to be used in mitigating oxidative-induced diseases in laying hens. Conversely, COS enhanced T-AOC and decreased MDA serum concentrations with no effect on antioxidant enzymes (T-SOD, CAT and GSH-Px) [19]. The discrepancies in the various studies may be attributable to physiological status, age, and duration of supplement feeding. 

The immunoglobulins (IgA, IgM, and IgY) are key immune indices denoting the immunity of the host [56]. Previous reports revealed that dietary FOS stimulated systemic responses in chickens by increasing the antibody titers of IgG and IgM [57]. Dietary XOS increased serum concentrations of both IgA and IgM in laying hens [58]. The enhanced immune response may be due to the capacity of prebiotics to stimulate immune response in birds [16]. Our findings are in agreement with previous reports that prebiotics increase secretion of immunoglobulins. In this study, the serum concentrations of the immunoglobulins (IgA, IgM, and IgG) were significantly improved in the feed additives group. IgA is produced by plasma cells in the lamina propria, secreted on the mucosal surface and predominant in intestinal secretions [59]. We speculate that the beneficial effect of FOS on mucosal integrity may have led to an increase in IgA secretion. The improvement in immunity may be linked with adequate bioavailability of circulating amino acids which is needed for immunocompetence [60] and dietary threonine and methionine enhances immunoglobulins synthesis [61]. Therefore, the key factor for improved immunoglobulins concentration could be the enhanced amino acid digestibility and utilization, facilitated by improved intestinal villi structures as more nutrients are utilized for immunoglobulin synthesis and not for gut cell renewal due to lower villi structures. Evidence has shown that the growth-promoting effect of prebiotics on lactic-acid-producing bacteria exerts an indirect influence on the immune system of the host [16,62]; thus, the prebiotic fermentation effect could be the key factor for enhanced immune response of the host. Nevertheless, dietary COS had no significant effect on serum immunoglobulin concentration of laying hens [19]. The variations may be due to age and environmental hygiene during the feeding trial. Furthermore, immune response compounds such as globulins and complement proteins which are capable of stimulating the immune system can be activated by immunostimulants [63]. Similar to our findings, COS increased the concentration of C3 proteins but had no effect on C4 [19]. The aforementioned findings provide enthralling evidence that the prebiotic FOS had immune-enhancing effects which provided an immune-stimulating response with significantly elevated plasma concentrations of IgA, IgG, and IgM.

Further analysis on the immunity of laying hens fed prebiotics using blood indices showed increased WBC and heterophils while no effect was observed on the H/L ratio. Similar to our findings, WBC was significantly higher than the control in birds fed COS [21,22]. The variations may be linked to environmental conditions employed during the study. The increased heterophils and reduced lymphocytes in birds fed 0.6% FOS may suggest that the birds are under stress, but the significant WBC may have reduced this effect and improved cellular immunity. The lack of an effect on H/L ratio may suggest better adaptation of the birds to the feed supplement. Improved animal health effectively promotes the development of various cells, tissues, and organs in the body of an animal.

### 4.4. Effect of Fructooligosaccharides on the Jejunal Villi Morphology of Laying Hens

Intestinal health often reflects the histomorphological structures which are mainly involved in modulating the capacity of animals to utilize nutrients. For example, broader villi surface area [64], lower epithelial thickness [65], increase in villi height and villi height to crypt depth [16] enhances nutrient absorption, whereas the decreased villi and deeper crypts reduce nutrient absorption [66]. Therefore, gut integrity provides allowance for efficient nutrient utilization which often results in improved egg production and egg quality. There is evidence that prebiotics enhanced intestinal morphology of birds: dietary XOS and inulin [31,58] and MDP [18] improved the jejunal villi heights, villi height to crypt depth ratio, and decreased crypt depths in laying hens. The enhanced intestinal villi structure could be linked to the competitive exclusion mechanism of prebiotics which will inhibit pathogenic microflora; thus, promoting intestinal epithelium nutrition [67]. Our findings affirm similar positive effects of prebiotics including FOS on jejunal villi development, evidenced by increased villi height, villi width, villi height to crypt depth ratio, and reduced crypt depth. The improved villi structure exerted a resultant positive effect on nutrient utilization, which accounts for most significant improvements in egg production and egg quality in this study. The improvement in villi structure may probably be due to the fact that butyric acid, which may result from gut fermentation of FOS, exerts a suppressive effect on intestinal pathogen invasion and inflammation; thus, improving the gut morphological structures. This claim is supported by the study of [68], which reported a positive effect of butyrate glyceride on epithelium integrity and structure of intestinal mucosa, leading to improved villi structures. Additionally, IgA is most abundant in the body, mainly involved in maintaining intestinal mucosal immunity and inhibition of intestinal infection [69]; thus, high synthesis of IgA in the study may have exerted a beneficial effect. Threonine, which is a component of intestinal mucin, controls the expression of biomarkers that are involved in intestinal development and GIT functioning [70,71], so high digestibility of threonine in this study may have facilitated high bioavailability of threonine. Additionally, prebiotics have been found to enhance synthesis of mucin, thereby improving the expression of jejunal mucin mRNA and the protective effect on epithelial cells of the intestine in broiler birds [72]. We infer that FOS may act in a like-manner in laying hens. However, there is contrary evidence that FOS had no effect on the ileal morphology of aged laying hens [14]. The discrepancies among studies may be due to the physiological status of the birds and age. We, therefore, deduce that the prebiotic fermentation effect, enhanced antioxidant capacity, and immune function, which culminated in protection of gut integrity from pathogens and oxidative stress, improved nutrient utilization in the gut and development of mucosal structures.

### 4.5. Effects of Fructooligosaccharides on the Apparent Fecal Digestibility of Amino Acids

Until recently, the influence of FOS on digestibility have gained less attention. This study is the first to report the effect of dietary FOS on apparent fecal amino acid digestibility in laying hens. In the current study, the fecal digestibility of crude protein and amino acids were significantly improved in the prebiotics group. Prebiotics possess antimicrobial properties and, FOS inclusive, have been reported to control inhabitation of pathogenic and beneficial bacteria species in the gut [73,74]. Thus, the reduced bacteria load in the feces with consequent improvement in fecal digestibility of nutrients may provide an explanation for the increased fecal CP and amino acid digestibility in birds. The higher digestibility of essential AA may be due to better crude protein digestibility; thus, there is less conversion of essential AA to non-essential purposes. The study of [75] reported that enhanced gut morphology reduces degradation of amino acids in the gastrointestinal tract. Therefore, the increased villi height, villi surface area, villi height to crypt depth ratio, and decreased crypt depth may have reduced amino acid degradation, leading to improved digestibility and reduced loss of amino acids in the feces. The improved amino acid digestibility is attributed to enhanced morphometric structure of the intestinal mucosa resulting from protective effects of antioxidant enzymes, immunoglobulins, and gut fermentation products.

### 4.6. Principal Component Analysis

The Principal Component Analysis (PCA) was explored in order to examine the relationship between the dietary treatments and determined parameters: amino acid digestibility, antioxidant and immune capacity, egg production, and laying performance (Figure 1). These plots showed distinct variations among the treatments with the parameters. Most of the parameters are dominant in the feed additive groups distinctively, lending more evidence that the improvements in egg quality and egg production are due to dietary prebiotics supplemented in the diets of the laying hens. Only the MDA content appears in the control, an implication that the treatments masked its effects in the feed additive group. There is a strong correlation between the diets and the output observed in the study.

## 5. Conclusions

Dietary fructooligosaccharides (FOS) enhanced performance and egg quality: egg mass, shell thickness, Haugh units, thick albumen content and height, while maintaining the physiological status as evidenced by improved antioxidant capacity, immune function, amino acid digestibility, and gut morphology. Furthermore, 0.6% of FOS had no adverse effect on the laying hens. This provides evidence that dietary FOS could be used as safe feed additives in poultry diets to produce high quality eggs. They could also serve as a substitute for antibiotics in promoting egg production and the health status of the animals.

## Figures and Tables

**Table 1:** The composition and nutrient levels of the basal and prebiotics-supplemented diets.

Ingredients	T1	T2	T3
Corn	63.4	62.79	62.15
Soybean meal	25.46	25.58	25.71
Oil	0.00	0.19	0.40
Stone powder	8.76	8.76	8.76
DL-methionine	0.18	0.18	0.18
Dicalcium phosphate	1.6	1.6	1.6
Salt	0.16	0.16	0.16
Premix (choline chloride)	0.25	0.25	0.25
FOS	0.0	0.3	0.6
Sodium sulfate	0.17	0.17	0.17
Phytase	0.02	0.02	0.02
Total	100	100	100
Nutrient Content	**%**		
Crude protein	16.50	16.50	16.50
Calcium	3.50	3.50	3.50
Total phosphorus	0.60	0.60	0.60
Available phosphorus	0.39	0.39	0.39
Metabolizable energy	11.23	11.23	11.23
SID methionine	0.434	0.434	0.433
SID lysine	0.796	0.796	0.798
SID tryptophan	0.176	0.176	0.176
SID threonine	0.560	0.560	0.561
SID methionine+ cysteine	0.653	0.652	0.651
SID isoleucine	0.666	0.666	0.667
SID cysteine	0.240	0.239	0.239
SID valine	0.746	0.746	0.746
SID arginine	1.030	1.031	1.033
SID leucine	1.414	1.412	1.410
SID serine	0.776	0.776	0.776
SID glycine	0.616	0.616	0.616

The values are calculated values. AME: apparent metabolizable energy. Vitamin and mineral premix provided the following per kg of diets: VA: 12,500 IU; VD3: 4125 IU; VE: 15 IU; VK: 2 mg; VB1: 1 mg; VB2: 8.5 mg; VB6: 8 mg; VB12: 5 mg; calcium pantothenate: 50 mg; niacin: 32.5 mg; biotin: 2 mg; folic acid: 5 mg; choline: 500 mg; Mn: 65 mg; I: 1 mg; Fe: 60 mg; Cu: 8 mg; Zn: 66 mg. SID—standard ileal digestibility; T1—basal diet; T2—basal diet + 0.3% FOS-fructooligosaccharides; T3—basal diet + 0.6% FOS-fructooligosaccharides.

**Table 2:** Effect of dietary fructooligosaccharides on performance of laying hens.

Items	Weeks	T1	T2	T3	SEM	*p*-Value
AEG (g)	1–4	61.42	60.58	60.89	0.89	0.359
	5–8	61.85	61.66	61.45	0.61	0.625
	9–12	59.98	60.10	60.51	0.68	0.489
	1–12	61.08	60.78	60.95	0.60	0.747
Egg mass (g)	1–4	58.33	58.09	59.20	0.98	0.213
	5–8	57.63	58.36	59.30	1.66	0.340
	9–12	58.83 ^b^	55.42 ^ab^	58.16 ^a^	1.91	0.017
	1–12	56.59 ^b^	57.29 ^ab^	58.90 ^a^	1.18	0.026
HDP %	1–4	93.33	94.39	94.89	1.33	0.730
	5–8	95.11 ^b^	95.93 ^ab^	97.22 ^a^	3.12	0.710
	9–12	89.75 ^b^	92.06 ^b^	96.13 ^a^	2.42	0.006
	1–12	92.73 ^b^	94.13 ^ab^	96.08 ^a^	1.51	0.014
Damaged eggs %	1–4	0.09	0.08	0.02	0.05	0.139
	5–8	0.10	0.05	0.10	0.06	0.355
	9–12	0.08	0.02	0.10	0.06	0.172
	1–12	0.09	0.03	0.02	0.05	0.139
Mortality rate %	1–4	0.01	0.00	0.00	0.00	0.391
	5–8	0.02	0.00	0.00	0.01	0.116
	9–12	0.01	0.00	0.00	0.00	0.391
	1–12	0.01	0.00	0.00	0.00	0.116
Feed intake (g)	1–4	116.58	119.80	119.18	3.30	0.435
	5–8	112.82	113.79	113.74	3.28	0.905
	9–12	121.18 ^c^	126.26 ^b^	132.41 ^a^	3.47	0.001
	1–12	116.86 ^b^	119.95 ^ab^	121.78 ^a^	2.09	0.013
FCR	1–4	2.00	2.06	2.01	0.06	0.364
	5–8	1.96	1.95	1.92	0.06	0.604
	9–12	2.25	2.28	2.28	0.11	0.911
	1–12	2.09	2.07	2.05	0.06	0.541

AEG—average egg weight; HDP—hen day-production; FCR—feed conversion ratio. Data represent the mean of six replicates of three hens each. Means within a row with different superscripts differ significantly (*p* < 0.05). SEM: Standard Error of Mean, T1—basal diet; T2—basal diet + 0.3% FOS-fructooligosaccharides; T3—basal diet + 0.6% FOS-fructooligosaccharides.

**Table 3:** Effect of dietary fructooligosaccharides on egg quality of laying hens.

Items	Weeks	T1	T2	T3	SEM	*p*-Value
Relative albumen weight %	1–4	60.15	59.08	60.59	1.28	0.29
	5–8	59.58 ^b^	60.75 ^b^	62.70 ^a^	1.26	0.01
	9–12	58.24 ^b^	63.50 ^a^	62.71 ^a^	1.26	0.01
	1–12	59.32 ^b^	61.11 ^a^	61.99 ^a^	0.36	0.02
Relative yolk weight %	1–4	29.01 ^a^	27.49 ^b^	28.21 ^ab^	0.69	0.02
	5–8	28.48 ^a^	28.10 ^a^	26.30 ^b^	0.72	0.01
	9–12	31.48 ^a^	27.54 ^b^	26.95 ^b^	0.61	0.01
	1–12	29.66 ^a^	27.71 ^b^	27.15 ^c^	0.27	0.01
Relative shell weight %	1–4	10.74	10.71	10.60	0.44	0.86
	5–8	10.82 ^a^	10.21 ^b^	10.12 ^b^	0.31	0.01
	9–12	10.31	10.29	10.24	0.53	0.98
	1–12	10.63	10.40	10.32	0.06	0.17
Shell thickness (mm)	1–4	44.88 ^b^	45.15 ^b^	47.03 ^a^	1.17	0.02
	5–8	46.88	47.05	46.94	0.70	0.93
	9–12	44.33 ^b^	45.80 ^ab^	46.13 ^a^	1.20	0.09
	1–12	45.37 ^b^	45.99 ^ab^	46.70 ^a^	0.21	0.03
Shell strength (N)	1–4	40.61	42.76	41.00	3.85	0.68
	5–8	45.40	45.71	47.53	3.40	0.63
	9–12	39.05	39.20	38.25	2.57	0.83
	1–12	41.69	42.55	42.25	0.44	0.74
Yolk color	1–4	5.72	5.61	6.11	0.68	0.56
	5–8	7.11	6.72	6.61	0.39	0.14
	9–12	5.72 ^b^	6.22 ^ab^	6.72 ^a^	0.47	0.02
	1–12	6.18	6.18	6.48	0.09	0.32
Albumen height (mm)	1–4	6.81	6.73	6.77	0.49	0.98
	5–8	7.80 ^b^	8.64 ^a^	8.66 ^a^	0.49	0.03
	9–12	6.19 c	7.89 ^b^	8.58 ^a^	0.36	0.01
	1–12	6.93 ^b^	7.75 ^a^	8.00 ^a^	0.08	0.01
Haugh units	1–4	80.23	77.92	78.83	3.55	0.62
	5–8	86.95	91.69	81.84	3.20	0.10
	9–12	76.16 ^c^	88.62 ^b^	95.16 ^a^	2.53	0.01
	1–12	81.11 ^c^	86.07 ^b^	88.60 ^a^	0.83	0.01
Thick-to-thin albumen ratio	1–4	1.00	1.20	1.13	0.14	0.13
	5–8	1.08	1.10	1.24	0.17	0.39
	9–12	1.14	1.49	1.75	0.14	0.01
	1–12	1.07	1.26	1.37	0.03	0.01

Data represent mean of six replicates of three hen each. Means within a row with different superscripts differ significantly (*p* < 0.05). SEM-Standard Error of Mean, T1—basal diet; T2—basal diet + 0.3% FOS-fructooligosaccharides; T3—basal diet + 0.6% FOS-fructooligosaccharides.

**Table 4:** Effects of fructooligosaccharides on antioxidant and immune response of laying hens.

Items	T1	T2	T3	SEM	*p*-Value
MDA (nmol/mL)	8.54 ^a^	3.5 ^b^	2.56 ^b^	0.83	0.01
CAT (U/mL)	9.67 ^c^	12.06 ^b^	15.295 ^a^	0.31	0.01
T-SOD (U/mL)	107.41 ^c^	130.89 ^b^	148.79 ^a^	5.88	0.01
T-AOC (U/mL)	11.50	12.20	14.32	2.27	0.17
GSH-Px (ng/mL)	53.45 ^b^	56.53 ^b^	78.61 ^a^	5.34	0.01
GST (ng/mL)	16.57 ^c^	17.45 ^b^	19.56 ^a^	0.59	0.01
IgG (ug/mL)	58.33 ^b^	61.68 ^b^	66.85 ^a^	5.67	0.093
IgM (ng/mL)	2372.50 ^b^	3935.00 ^a^	4080.83 ^a^	392.70	0.01
IgA (ng/mL)	4762.78 ^b^	5471.11 ^a^	5918.33 ^a^	335.71	0.01
C3 (mg/mL)	0.073 ^b^	0.081 ^ab^	0.085 ^a^	0.01	0.05
C4 (mg/mL)	0.044	0.045	0.045	0.00	0.86

MDA—malondialdehyde; CAT—catalase; T-SOD—total superoxide dismutase; T-AOC—total antioxidant capacity; GSH-Px—glutathione peroxidase; GST—glutathione transferase; IgG—immunoglobulin G; IgM—immunoglobulin M; IgA—immunoglobulin A; C3 and C4—complement proteins. Data represent mean of six replicates of three hens each. Means within a row with different superscripts differ significantly (*p* < 0.05). SEM-Standard Error of Mean, T1—basal diet; T2—basal diet + 0.3% FOS-fructooligosaccharides; T3—basal diet + 0.6% FOS-fructooligosaccharides.

**Table 5:** Effects of fructooligosaccharides on hematology indices of laying hens.

Items	T1	T2	T3	SEM	*p*-Value
WBC (×10^9^/L)	12.1 ^c^	19.97 ^a^	16.54 ^b^	1.933	0.01
RBC (×10^12^/L)	2.26	2.31	2.42	0.138	0.22
Hb (g/L)	71	73.3	74.667	5.209	0.57
PCV (%)	35	35.5	37.65	2.26	0.24
MCV (fL)	155	154	155.72	4.923	0.82
MCH (Pg)	31.4	31.8	30.917	1.588	0.72
MCHC (g/L)	203	207	198.33	5.407	0.10
Platelets (×10^9^/L)	11.7	12	12	2.49	0.97
Heterophil (×10^9^/L)	6.25 ^b^	9.9 ^a^	8.555 ^ab^	1.87	0.03
Lymphocytes (×10^9^/L)	5.19 ^b^	7.18 ^a^	4.6367 ^b^	1.338	0.02
H/L	1.34	1.45	1.97	0.35	0.25
Monocyte (×10^9^/L)	0.16	1.15	0.80	0.517	0.12
Eosinophil (×10^9^/L)	0.05	0.32	0.27	0.244	0.28
Basophil (×10^9^/L)	0.98	1.42	2.25	0.927	0.17

WBC—white blood cells count; RBC—red blood cells count; Hb—hemoglobin count; PCV—packed cell volume; MCV—mean corpuscular volume; MCH—mean corpuscular hemoglobin; MCHC—mean corpuscular hemoglobin concentration; H/L—heterophil/lymphocyte. Data represent mean of six replicates of three hens each. Means within a row with different superscripts differ significantly (*p* < 0.05). SEM-Standard Error of Mean, T1—basal diet; T2—basal diet + 0.3% FOS-fructooligosaccharides; T3—basal diet + 0.6% FOS-fructooligosaccharides.

**Table 6:** Effects of fructooligosaccharides on jejunal villi morphometrics of laying hens.

Items	T1	T2	T3	SEM	*p*-Value
VH	938.80 ^b^	1199.45 ^a^	1298.98 ^a^	38.20	0.000
VW	141.20 ^b^	165.08 a	155.91 ^ab^	4.36	0.070
CD	155.60 ^a^	118.65 ^b^	117.18 ^b^	5.74	0.002
VH:CD	6.35 ^b^	10.36 ^a^	8.55 ^a^	0.53	0.002
VSA	0.43 ^b^	0.62 ^a^	0.65 ^a^	0.02	0.001

VH—villi height (μm); VW—villi width (μm); CD—crypt depth (μm); VH:CD—villi height to crypt depth ratio; VSA—villi surface area (mm2) = 2π × (VW/2) × VH. Means within a row with different superscripts differ significantly (*p*< 0.05) SEM-Standard Error of Mean, T1—basal diet; T2—basal diet + 0.3% FOS-fructooligosaccharides; T3—basal diet + 0.6% FOS-fructooligosaccharides.

**Table 7:** Effects of fructooligosaccharides on apparent fecal amino acid digestibility of laying hens.

Items (%)	T1	T2	T3	SEM	*p*-Value
Crude protein	59.20 ^b^	69.24 ^a^	71.98 ^a^	5.26	0.01
Asparagine	75.29 ^b^	79.05 ^ab^	81.23 ^a^	3.05	0.03
Threonine	69.48 ^b^	72.79 ^ab^	76.73 ^a^	3.83	0.03
Serine	77.63 ^b^	79.45 ^ab^	83.41 ^a^	3.07	0.03
Glutamine	84.57 ^b^	87.17 ^ab^	88.62 ^a^	2.08	0.03
Proline	80.42	81.93	86.43	4.30	0.11
Glycine	−3.14 ^b^	4.96 ^ab^	23.71 ^a^	15.48	0.05
Alanine	65.77	69.73	71.22	5.00	0.24
Cysteine	73.58	76.54	76.51	3.72	0.38
Valine	73.91	78.12	77.34	3.72	0.21
Methionine	83.01 ^c^	90.12 ^a^	85.96 ^b^	2.09	0.01
Met + Cys	79.28 ^b^	85.75 ^a^	82.34 ^ab^	2.62	0.01
Isoleucine	74.16 ^b^	79.33 ^a^	79.23 ^a^	3.47	0.06
Leucine	80.21	83.28	83.43	2.71	0.14
Tyrosine	80.54 ^b^	85.78 ^a^	85.69 ^a^	3.71	0.08
Phenylalanine	89.25	92.55	90.69	2.55	0.18
Histidine	51.02	62.59	52.71	10.76	0.26
Lysine	73.19 ^b^	77.87 ^a^	79.27 ^a^	3.30	0.03
Arginine	83.97	85.65	86.89	1.98	0.10
Tryptophan	73.55 ^b^	85.17 ^a^	83.93 ^a^	2.94	0.01

Met + Cys—methionine cysteine. Data represent mean of six replicates of three hens each. Means within a row with different superscripts differ significantly (*p* < 0.05). SEM-Standard Error of Mean, T1—basal diet; T2—basal diet + 0.3% FOS-fructooligosaccharides; T3—basal diet + 0.6% FOS-fructooligosaccharides.

## Data Availability

All data included in this study are available by contacting the corresponding author. Data is contained within the article.
